# Bacteriology

**Published:** 1898-09

**Authors:** T. E. Carmody


### THE DENTAL REGISTER.

VOL. LII.] SEPTEMBER, 1898. [No. 9.

### COMMUNICATIONS.

### Bacteriology.

BY T. E. CARMODY, D D.S.

A Thesis for the Degree of Doctor of Dental Science, University of Michigan, Dental Department.

In this paper it will be my object to give the results of experiments undertaken to determine the relative value of some of the antiseptics used in dentistry.

First, what is an antiseptic and what are its uses in dentistry?

An antiseptic is a substance which when brought in contact with micro-organisms prevents their growth, but does not destroy them.

The uses of antiseptics in dentistry are various and the drug must be adapted to the use for which it is intended.

In all of these experiments we have spared no effort to eliminate all sources of error, and although some errors have probably been overlooked, we trust they are few. In order to have the media even it has all been prepared with greatest care. Great care has also been taken in the growth of the germs. After the media were prepared and sterilized, all the tubes were placed in the incubator for some days; and if growth had taken place in any of them they were discarded and only those which were known to be steril were used.

The methods of determining the power of drugs are as various as their uses. The first method employed was to determine the antiseptic power of some of the more common antiseptics on micro-organisms grown in beef broth.

Take some freshly prepared sterile beef broth and to this add a few drops of a fresh culture ( twenty-four hours old), then place in four large test tubes the following amounts: in the first 9 c.c., in the second 9.5 c.c., in the third 9.75 c.c., and in the fourth 9.9 c.c. Then to each of these add a sufficient amount of the drug to be tested in solution to make 10 c.c. These were set aside in the incubator for twenty-four hours, the results noted, and returned to the incubator for another twenty-four hours. Then if growth had not taken place in all the tubes, it was carefully noted in which one it had, and how the growth compared with that in the control both as to macroscopic and microscopic appearances.

By this method we could tell proximately the range of antiseptic power of a substance in question. The following are the results:

FORMALIN.

| | | |
|---|---|---|
| 1. | 1- 1,000 | No growth. |
| 2. | 1- 2,000 | No growth. |
| 3. | 1- 4,000 | Slight growth, single and pairs. |
| 4. | 1-10,000 | Quite heavy growth, single and pairs, small bunches. |

SODIUM BENZOATE.

| | | |
|---|---|---|
| 1. | 1- 1,000 | Slight growth, single and pairs. |
| 2. | 1- 2,000 | Slight growth, single and pairs, short chains. |
| 3. | 1- 3,000 | Quite heavy growth, single and pairs, short chains. |
| 4. | 1-10,000 | Heavy growth, single and pairs, all bundles. |

MERCURIC CHLORIDE.

| | | |
|---|---|---|
| 1. | 1- 5,000 | No growth. |
| 2. | 1-10,000 | No growth. |
| 3. | 1-20,000 | Quite heavy growth, bunches, short chains and single. |
| 4. | 1-50,000 | Heavy growth, bunches, short chains and single. |

PENROL.

| | | |
|---|---|---|
| 1. | 1- 1,000 | Slight growth, small bunches and scarce. |
| 2. | 1- 2,000 | Slight growth, small bunches and chains. |
| 3. | 1- 4,000 | Quite heavy growth, single and chains. |
| 4. | 1-10,000 | Heavy growth, single, chains and bunches. |

The next experiment was for the purpose of testing the disinfectant power and was performed in the following manner:

Ten c.c. of a fresh bouillon were placed in each of several large sterile test tubes and sterilized in usual way. Then to 105 c.c. of sterile water in sterile beaker add the germ to be tested and thoroughly whip up if necessary, then warm gently to get complete separation of cells. Then to remove agglomerations of cells filter through a sterile glass wool filter and receive in sterile beaker.

A control agar plate was made and this allowed to remain in incubator for some days then removed and colonies counted.

In a small sterile Erlengmyer flask place 25 c.c. of solution to be tested; add an equal volume of the suspension and mix well. This dilutes your solution one-half, so that it should not be tested in as weak a solution as that of which you wish to get the action.

Direct Method.—At intervals of 5, 10, 15, 30, 45 and 60 minutes, transfer a small loop full of this mixture to tube of beef broth. Label and set aside at 37° for some days; at same time transfer a loop to melt agar at 45°; mix and pour into Petri dish. Set aside at 37° and count colonies.

$H_2S$ Method.—At intervals above mentioned transfer $2\frac{1}{2}$ c.c. of the mixture to a sterile Liborous tube containing an equal volume of sterile thirty percent salt solution and calcium carbonate. Pass $H_2S$ for five minutes, mix well, and let stand for five to ten minutes pass $H_2S$ to deposit, then transfer two loops of this mixture to beef broth and place at 37° C. Transfer two loops to tubes of melted agar at 45°, plate, and set aside at 37° and count colonies.

This method is used only with mercuric chloride solutions as the $H_2S$ precipitates mercury.

Table shows results obtained in testing the germicidal effect of chemicals on Sanarelli's germ:

| | 5 min. | 10 min. | 15 min. | 30 min. | 45 min. | 60 min. | |
|---|---|---|---|---|---|---|---|
| $HgCl_2$ | ...... | ...... | ...... | ...... | ...... | ...... | 48 hours |
| 1-1,000 | ...... | ...... | ...... | ...... | ...... | ...... | 72 " |
| $HgCl_2$ + | ...... | ...... | ...... | ...... | ...... | ...... | 48 " |
| $H_2S$ | ...... | ...... | ...... | ...... | ...... | ...... | 72 " |

| | 5 min. | 10 min. | 15 min. | 30 min. | 45 min. | 60 min. | |
|---|---|---|---|---|---|---|---|
| Carbolic | ...... | ...... | ...... | ...... | ...... | ...... | 48 hours |
| acid 5 percent | ...... | ...... | ...... | ...... | ...... | ...... | 72 " |
| Formalin | ...... | ...... | ...... | ...... | ...... | ..... | 48 " |
| 2 percent | ...... | ...... | ...... | ...... | ...... | ...... | 72 " |

| | 5 min. | 10 min. | 15 min. | 30 min. | 45 min. | 60 min. |
|---|---|---|---|---|---|---|
| $HgCl_2$ 1-1,000 | 0 | 1 | 0 | 0 | 0 | 7 |
| $HgCl+H_2S$ | 0 | 0 | 2 | 2 | 5 | 1 |
| Formalin | 0 | 0 | 1 | 0 | 0 | 0 |
| Carbolic acid | 5 | 0 | 1 | 0 | 0 | 0 |
| Control | 2200 | | | | | |

The following table shows the disinfectant action on Havelburg's germs.

| | 5 min. | 10 min | 15 min. | 30 min. | 45 min. | 60 min. |
|---|---|---|---|---|---|---|
| $HgCl_2$ | ...... | ...... | ...... | ...... | ...... | ...... |
| 1-1,000 | ...... | ...... | ...... | ...... | ...... | ...... |
| $HgCl_2$ + | ...... | ...... | ...... | ...... | ...... | ...... |
| $H_2S$ | ...... | ...... | ...... | ...... | ...... | ...... |
| Formalin | ...... | ...... | ...... | ...... | ...... | ...... |
| 1 percent | ...... | ...... | ...... | ...... | ...... | ...... |
| Carbolic | ...... | ...... | ...... | ...... | ...... | ...... |
| acid 2 percent | ...... | ...... | ...... | ...... | ...... | ...... |

The following table shows the number of colonies of Havelburg's germ grown on agar plates.

The first counting is given on first line. The plates were put back again and counted later to show inhibiting power of chemicals.

| | 5 min. | 10 min. | 15 min. | 30 min. | 45 min. | 60 min. | |
|---|---|---|---|---|---|---|---|
| $HgCl_2$ | 8 | 2 | 1 | 0 | 0 | 0 | 24 hours |
| 1-1,000 | 8 | 4 | 1 | 2 | 2 | 3 | |
| $HgCl_2$ + | 105 | 2 | 6 | 8 | 0 | 0 | 24 " |
| $H_2S$. | 155 | 21 | 6 | 10 | 1 | 0 | |
| Formalin | 1 | 0 | 0 | 1 | 0 | 0 | 24 " |
| 2 percent | 50 | 1 | 2 | 0 | 0 | 0 | |
| Carbolic | 1 | 0 | 0 | 0 | 0 | 0 | 24 " |
| acid 2 percent | 1 | 1 | 0 | 0 | 0 | 0 | |
| Control | 3,700 | | | | | | 24 " |

The experiments with Havelburg's germ were performed in exactly the same manner as those in which Sanarelli's germ was used.

Experiments were also preformed to determine the effect of heat on micro-organisms which are as follows:

First, Moist Heat.—As in the former experiment it was necessary to use every precaution to have the suspension free from masses of germs, in order to tell the exact thermal death point, for in this way the germs will attain the temperature of the surrounding medium in all parts at the same time or proximately the same. For this reason the beef broth culture should first be diluted with sterile water, then filtered through a sterile glass wool filter and received in sterile cylinder. Then small amounts are drawn into sterile capillary tubes and the end sealed, also sealed above liquid so that you have several small tubes about three inches in length.

Then a large boilerfull of water is heated until a constant temperature of 50° is attained. For this purpose a thermo-regulator is required. A small wire support is placed in the water and when temperature is again found to be constant the tubes are placed on the support. Watch the temperature closely so that no variation takes place. At the end of 15, 30, 45 and 60 minutes remove and immerse at once in cold water. When cool remove and wipe dry with sterile cloth. Then nick one end of the tube with a file, break off and pass it through a flame two or three times, to slerilize, being careful not to place too much of the tube in flame as the micro-organisms may be affected by it and thus the experiment would prove nothing. Then place the open end in sterile beef broth tube and heat the closed end in flame; the liquid is forced into the bouillon tube. Also place four at 60° and remove at the end of 1, 5, 10 and 15 minutes.

At 70° in same manner four are placed and removed at the end of 1, 3, 5 and 8 minutes. Also at 100° and removed at the end of 1, 3 and 5 minutes.

Add a small drop of original suspension to each of three bouillon tubes and expose at 100° for 1, 3 and 5 minutes. Remove, cool at once and place in incubator. Also in each of three tubes place a short silk thread impregnated with germs and expose exactly as above and compare results.

Action of Moist Heat on Streptococcus Pyogenes.

| | | | | |
|---|---|---|---|---|
| 50° | 15 min. | 30 min. | 45 min. | 60 min. |
| | ... | ... | ... | ... |
| 60° | 1 min. | 5 min. | 10 min. | 15 min. |
| | ... | ... | ... | ... |
| 70° | 1 min. | 3 min. | 5 min. | 8 min. |
| | 0 | 0 | 0 | 0 |
| 100° | 1 min. | 3 min. | 5 min. | |
| Capillary tube | 0 | 0 | 0 | |
| Boullon tube | 0 | 0 | 0 | |

It can be seen by the table that the lower tempatures affected the germs only for a short time as growth took place later, but the higher temperatures killed.

Second, Dry Heat.—Ten short silk threads were saturated with a filtered suspension and then allowed to dry at 35° in a Petri dish. Then four of these were placed in sterile Esmark dish in hot air sterilizer regulated at 100°.

These were removed rapidly at the end of 30, 45, 60 and 80 minutes and placed in tubes of sterile bouillon. They must be removed rapidly because if door of sterilizer is allowed to remain open any great length of time the temperature will fall far below the point you wish to maintain, therefore this would be a source of error. Place three of these at 120° and remove at the end of 15, 30 and 45 minutes. Also two at 140° and remove at the end of 5 and 15 minutes.

These tubes were then placed in incubator and for two or three days changes noted.

In this experiment the streptococcus pyogenes was used suspended same as in experiment with moist heat and the threads were allowed to remain in this solution long enough to become thoroughly impregnated with the germ.

Effect of Dry Heat on Streptococcus Pyogenes.

| | | | | |
|---|---|---|---|---|
| 100° | 30 min. | 45 min. | 60 min. | 1½ hrs. |
| | ... | 0 | 0 | 0 |

| | | | |
|---|---|---|---|
| 120° | 15 min. | 30 min. | 45 min. |
| | ... | 0 | 0 |
| 140° | 5 min. | 15 min. | |
| | 0 | 0 | |

All of the experiments thus far have been with bouillon suspension of one kind of germ. We next tried experiments which have somewhat more practical value to the dentist because in all the following experiments fresh saliva was used which gave us a greater number of kinds of germs and therefore gave a more severe test.

The sliva used was always perfectly fresh and in each experiment a different sample of saliva was used unless the experiment failed, in which case it was simply repeated, using the same saliva.

The first method employed was as follows:

The saliva required was collected in sterile Esmark dish and filtered through sterile glass wool filter and received in sterile tube. Then .5 c.c. of saliva was placed in sterile Esmark dish and an equal volume of the antiseptic added and thoroughly mixed. Then at the end of 2½, 5, 10 and 15 minutes, one loop was transferred to melted agar at 45°, the lip of tube sterilized and agar poured into sterile Petri dish. These dishes were placed in incubator for twenty-four hours, removed, colonies counted and returned for another twenty-four hours.

It will be noticed in the following tables that any one antiseptic is not equally good for all persons to use as a mouth wash.

It must be remembered that the number of colonies represent the number of germs in the transplanted loop, which were not killed, although they may have been inhibited.

Experiment No. 1.

| Control—1043. | 2½ min. | 5 min | 10 min. | 15 min. |
|---|---|---|---|---|
| $HgCl_2$ 1–2,000 | 143 | 81 | 75 | 20 |
| Lysol, 2 percent | 0 | 13 | 0 | 26 |
| Listerine (full strength) | 4 | 6 | 29 | 3 |
| Euthymol (full strength) | 0 | 3 | 0 | 36 |
| Miller's 1–7 | 0 | 1 | 0 | 2 |
| Borolyptol (full strength) | 500 | 480 | 630 | 475 |
| Form-aseptol (full strength) | 36 | 38 | 34 | 2 |

EXPERIMENT No. 2.

| Control—432. | $2\frac{1}{2}$ min. | 5 min. | 10 min. | 15 min. |
|---|---|---|---|---|
| $HgCl_2$ 1-1,000 | 400 | 235 | 228 | 84 |
| Lysol, 2 percent | 40 | 0 | 0 | 0 |
| Listerine | 1 | 2 | 1 | 1 |
| Euthymol | 0 | 0 | 0 | 0 |
| Millers's, 1-7 | 220 | 80 | 8 | 7 |
| Borolyptol | 200 | 130 | 140 | 95 |
| Form-aseptol | 100 | 5 | 1 | 75 |
| Formalin, 1 percent | 750 | 54 | 67 | 36 |

EXPERIMENT No. 3.

| Control—3,600 | $2\frac{1}{2}$ min. | 5 min. | 10 min. | 15 min. |
|---|---|---|---|---|
| $HgCl_2$ 1-1,000 | 360 | 540 | 1,380 | 100 |
| Lysol, 2 percent | 2 | 1 | 1 | 3 |
| Listerine | 0 | 3 | 0 | 0 |
| Euthymol | 6 | 40 | 9 | 5 |
| Miller's, 1-7 | 24 | 7 | 27 | 13 |
| Borolyptol | Uncountable | 2,876 | 2,220 | 1,500 |
| Form-aseptol | 5 | 480 | 300 | 1 |
| Formalin, 1 percent | 570 | 2 | 7 | 25 |
| Carbolic, 2 percent | 18 | 2 | 7 | 25 |
| Sanitol, 1-2 | 1 | 1 | 11 | 1 |

EXPERIMENT No. 4.

| Control—21,760 | $2\frac{1}{2}$ min. | 5 min. | 10 min. | 15 min. |
|---|---|---|---|---|
| $HgCl_2$ 1-1,000 | 600 | 300 | 150 | 200 |
| Lysol, 2 percent | 1 | 1 | 0 | 5 |
| Listerine | 49 | 18 | 0 | 7 |
| Euthymol | 1 | 128 | 352 | 6 |
| Miller's, 1-7 | 600 | ...... | 8 | 7 |
| Borolyptol | 600 | 540 | 304 | 216 |
| Form-aseptol | 200 | 14 | 16 | 3 |
| Formalin, 1 percent | 130 | 580 | 25 | 40 |
| Sanitol | 27 | 2 | 1 | 1 |

EXPERIMENT No. 5.

| Control—2,400 | $2\frac{1}{2}$ min. | 5 min. | 10 min. | 15 min. |
|---|---|---|---|---|
| $HgCl_2$ 1-1,000 | 100 | 3 | 15 | 4 |
| Lysol, 1 per cent | 1 | 0 | 1 | 0 |

| Control—2,400 | 2½ min. | 5 min. | 10 min. | 15 min. |
|---|---|---|---|---|
| Listerine | 5 | 0 | 1 | 0 |
| Euthymol | 0 | 0 | large | 0 |
| Miller's, 1-7 | 1 | 0 | 0 | 0 |
| Borolyptol | 2,280 | 1,560 | 1,380 | 1,000 |
| Form-aseptol | Uncountable | 7 | 6 | 4 |
| Formalin | 34 | 1 | 53 | 4 |
| Carbolic, 2 percent | Unc. | Unc. | 128 | 0 |
| Sanitol | 8 | 0 | 1 | 0 |

EXPERIMENT No. 6.

| Control—2,220. | 2½ min. | 5 min. | 10 min. | 15 min. |
|---|---|---|---|---|
| Sanitol, | 0 | 0 | 27 | 0 |
| Lysol—2 percent, | 25 | 0 | 0 | 0 |
| Listerine, | 0 | 2 | 2 | 0 |
| Euthymol, | 2 | 0 | 0 | 0 |
| Miller's—1-7, | 1 | 2 | 0 | 0 |
| Borolyptol, | 960 | 1,200 | 720 | 420 |
| Form-aseptol, | 300 | 0 | 0 | 0 |
| Formalin—1 percent, | 0 | 0 | 2 | 12 |
| Carbolic—2 percent, | 1,500 | 1,800 | 1,320 | 540 |

EXPERIMENT No. 7.

| Control—1,680. | 2½ min. | 5 min. | 10 min. | 15 min. |
|---|---|---|---|---|
| $HgCl_2$—1-1,000, | 0 | 2 | 0 | 0 |
| Listerine, | 3 | 3 | 1 | 1 |
| Euthymol, | 3 | 1 | 1 | 7 |
| Borolyptol, | 300 | 0 | 123 | 15 |
| Form-aseptol, | 3 | 0 | 0 | 0 |
| Sanitol, | 1 | 0 | 1 | 6 |
| Formalin—1 percent, | 59 | 34 | 6 | 2 |

The next set of experiments performed were to test the power of the essential oils. On account of their insolubility in water solutions were made in absolute alcohol of four parts of alcohol to one of oil.

Then the experiment was carried out in the same manner as for the mouth washes, but it was found that the oil was thrown out of solution when brought in contact with saliva, and we simply had an emulsion. Although this was a great objection it was not the chief one, for it was found that growth did not take place in the alcohol control, and therefore the alcohol must have some action or there must be some mistake. Thinking the latter to be the case, the experiment was repeated several times with no better success. Then, thinking the laboratory alcohol might have been tampered with, and contain some antiseptic, we first secured some absolute alcohol from another source. This gave the same results, and another sample was obtained and tried on staphylococcus and streptococcus with no growth, then anthrax, eberth, coli and friedlander were tried with no growth after five minutes, except of friedlander.

In performing these experiments with alcohol the alcohol was diluted with water down to 80 percent, thus making the same amount of alcohol as in the essential oil solutions, and then equal parts of this and saliva or germ suspension were taken, thus diluting it to 40 percent.

The following tables will explain themselves:

EXPERIMENT No. 1.

| Control—3,600. | $2\frac{1}{2}$ min. | 5 min. | 10 min. | 15 min. |
|---|---|---|---|---|
| Alcohol, | 12 | 7 | 0 | 0 |

EXPERIMENT No. 2.

| Control—4,500. | $2\frac{1}{2}$ min. | 5 min. | 10 min. | 15 min. |
|---|---|---|---|---|
| Alcohol, | 24 | 19 | 2 | 0 |

EXPERIMENT No. 3.

| Control—20,000. | $2\frac{1}{2}$ min. | 5 min. | 10 min. | 15 min. |
|---|---|---|---|---|
| Alcohol, | 100 | 31 | 9 | 0 |

EXPERIMENT No. 4.

| Control—1,500. | $2\frac{1}{2}$ min. | 5 min. | 10 min. | 15 min. |
|---|---|---|---|---|
| Alcohol, | 0 | 21 | 10 | 3 |

EXPERIMENT No. 5.

| Control—16,000. | $2\frac{1}{2}$ min. | 5 min. | 10 min. | 15 min. |
|---|---|---|---|---|
| Alcohol, | 70 | 24 | 2 | 0 |

EXPERIMENT No. 6.

| Control—3,000. | $2\frac{1}{2}$ min. | 5 min. | 10 min. | 15 min. |
|---|---|---|---|---|
| Alcohol, | 5 | 1 | 0 | 0 |

EXPERIMENT NO. 7. PUS GERMS.

| Control—abundant. | $2\frac{1}{2}$ min. | 5 min. | 10 min. | 15 min. |
|---|---|---|---|---|
| Alcohol, | 2 | 1 | 0 | 0 |

EXPERIMENT NO. 8. ALCOHOL.

| | Anthrax. | Eberth. | Coli. | Friedlander. |
|---|---|---|---|---|
| Control, | 420 | Abundant. | 12,000 | 3,000 |
| $2\frac{1}{2}$ min., | 0 | 1 | 0 | 50 |
| 5 min., | 2 | 0 | 1 | 24 |
| 10 min., | 1 | 2 | 0 | 5 |
| 15 min., | 6 | 0 | 0 | 0 |

With anthrax there was growth after fifteen minutes' exposure. This was probably due to resistant spores.

In conclusion it would be well to state that some of the antiseptics and disinfectants that appear to be very good in laboratory are not as good practically because of other properties which they have. For example, $HgCl_2$ appears to be very good from laboratory experiments for a mouth wash, but because of its affinity for albumin it loses its power very quickly, and what is left has not the power of penetrating the precipitate formed. Another and more serious objection is that it injures the tooth structure.

Although carbolic acid precipitates albumin, it has the power of penetrating the precipitate when formed, therefore it is very much better, but it has an objectionable taste and odor.

Although formalin has none of the objectionable properties of the former two it is very irritating, and if used in effective solutions it destroys tissue, hence makes fresh field for infection.

Borolyptol, although of pleasant odor and taste, is absolutely worthless as an antiseptic.

Miller's mouth wash is not as objectionable as the others and is a good antiseptic. (The formula used is the one which does not contain mercuric chloride.)

Form-aseptol is also good, and along with listerine and euthymol is among the best proprietary preparations, but as these were used in full strength, allowance must be made.

Although we would be able to tell little about antiseptics used if it were not for laboratory experiments, we must remember also the therapeutics of substances used, and not use something on the body for an antiseptic or disinfectant that will be so intensely irritating as to do more harm than good. Neither should we discard something that is non-irritating, but which has given good results because laboratory experiments do not prove it to be up to the standard.

